# Advances on Measuring Deep-Seated Ground Deformations Using Robotized Inclinometer System

**DOI:** 10.3390/s20133769

**Published:** 2020-07-05

**Authors:** Paolo Allasia, Danilo Godone, Daniele Giordan, Diego Guenzi, Giorgio Lollino

**Affiliations:** Italian National Research Council, Research Institute for Hydrogeological Prevention and Protection, Geohazard Monitoring Group (GMG) Strada delle Cacce, 73, 10135 Torino, Italy; paolo.allasia@irpi.cnr.it (P.A.); daniele.giordan@irpi.cnr.it (D.G.); diego.guenzi@irpi.cnr.it (D.G.); giorgio.lollino@irpi.cnr.it (G.L.)

**Keywords:** inclinometer, landslides, monitoring systems, subsoil observation, geo-engineering

## Abstract

In the field of geo-hazards and geo-engineering, monitoring networks represent a key element for the geological risk assessment and the design and management of large infrastructures construction. In the last decade, we have observed a strong development on remote sensing techniques but just small changes in the subsoil observations. However, this type of measurement is very important to have a three-dimensional representation of the studied area, since the surface measurements often represent a sum of deformations that develop in a complex way in the subsoil. In this paper, we present a robotic inclinometer system developed to acquire deep-seated ground deformations in boreholes. This instrumentation combines advantages offered by manual inclinometer measurements with a robotized approach that improves the results in term of accuracy, revisiting time, and site accessibility. The Automated Inclinometer System (AIS) allows one to explore automatically all the length of the monitored borehole using just one inclinometer probe with a semi-wireless system. The paper presents the system and a detailed dataset of measurements acquired on three inclinometer tubes installed for the monitoring of the construction phase of the new Line C Metro of Rome. The dataset was acquired in real monitored site and undisturbed conditions and can represent a benchmark for modern inclinometer measurements.

## 1. Introduction

**Figure 1:**
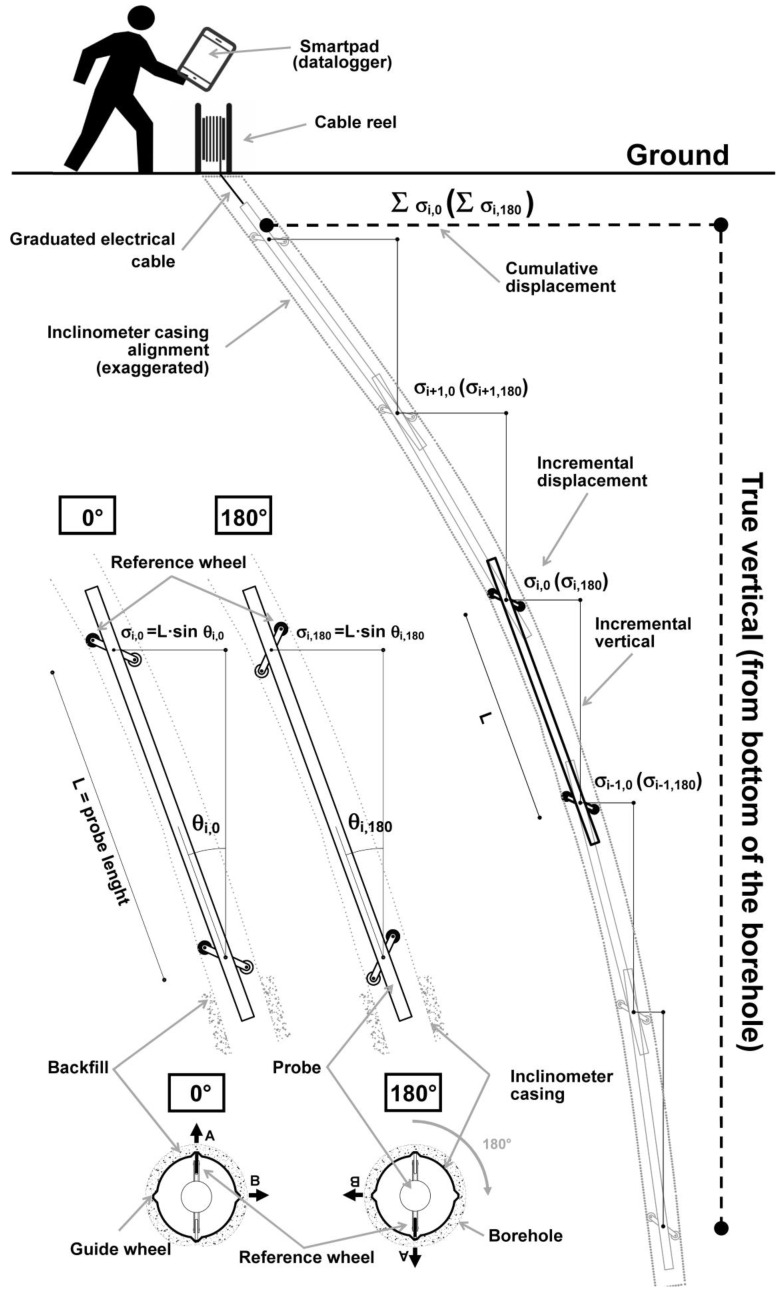
Measurement of horizontal deep-seated ground deformation using inclinometer probe.

**Figure 2:**
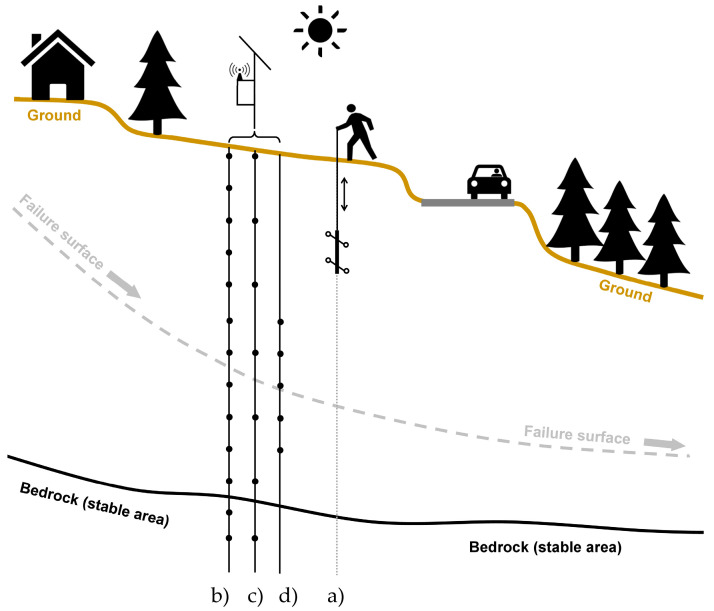
Standard approach to the deep-seated horizontal ground deformation. (**a**) Manual measurements with operator and portable probe; (**b**) In-Place Inclinometer (IPI) with high number of sensors for all the length of the borehole; (**c**) IPI with reduced number of sensors for all the length of the borehole; and (**d**) IPI with a reduced number of sensors only for a small part of the borehole (close to the hypothesized failure surface).

The monitoring of soil and subsoil deformation phenomena plays an important role in the fields of earth sciences and engineering-geology. Currently available instruments and techniques allow one to track the evolution of geo-hydrological events and the construction of large infrastructure with high accuracy and temporal resolution. In the last decade, a huge development in the methodologies to measure ground deformation was observed. On the contrary, at the same time, only a reduced increase in the field of subsoil deformation measurement techniques was recorded. This difference is mainly related to the significant development in the field of remote sensing and its possibility to observe large areas in ever-shorter times [1,2,3,4]. However, the deformation measured at the ground level represents a sum of what happens in the subsoil. In many cases, in order to explain the real mechanism that caused the surficial deformation measured, a series of hypotheses is needed. In this context, the measurement of subsoil deformations represents an important source of information for a more correct and more complete interpretation of the phenomenon in terms of thickness of soil involved and vertical distribution of stress and resistant forces. Despite the importance of these parameters, in the last decade, the methods and instrumentation to measure subsoil deformations are quite unchanged [5,6,7,8,9,10,11,12,13,14,15]. The main technique used is the measurement of inclination over the time in specific boreholes (i.e., inclinometers). The observed phenomena (landslides, geotechnical works, etc.) induce a shape deformation into the borehole and these deformations are measured in terms of the inclination variation (Figure 1 and Figure 2). This measurement, conceived in the ’50s by Wilson [5,9], is carried out using an inclinometer probe, which is progressively positioned by an operator at the designed depth, usually every 50 cm for all the length of the borehole [5,9,10].

In order to automatize these measurements, in the early ‘90s [5], inclinometer sensor chains (In Place Inclinometers—IPI) were used. Chains are permanently placed inside the inclinometer tube every, approximately, one meter (or multiple). These systems are installed for the entire length of the borehole or only in some part of this (in the most active or in particular sections). With this system, it was possible to describe the evolution of the phenomenon in time but with some critical issues related to vertical discretization, the long-time sensors drift and the high costs especially for long boreholes (Figure 2, Table 1). Regarding the general accuracy of an inclinometric measurement, former studies and technical literature [5,9,10,12,13] provided some suggestions that, over time, they were used as a standards and best practice rules [15,16,17]. The recent spread of the low cost Micro Electro-Mechanical Systems (MEMS) technology combined with the increasing demand for higher temporal resolutions pushed an important development of the In-Place Inclinometer (IPI) systems with a general cost reduction [18,19,20,21,22,23], However, the overall cost per measuring element remains medium/high due to the cost of the support structure and interconnection between each element (steel, carbon, etc.). Even today, to reduce the costs, only a minimum number of sensors is installed or with an increasing distance between them (Figure 2c) or with a partial installation only in the alleged most active sectors of movement (Figure 2d). This approach features a series of critical issues mainly related to the incomplete discretization of the borehole and the inability to identify movements in the non-instrumented sectors. To reduce these critical issues it is recommended, at least once per year, to extract the string of sensors and make a complete manual measurement with portable inclinometer system (Figure 2a). This operation is not always easily feasible due to the weight of the sensors chain, the strong tube-string coupling or in the case of large deformations of the inclinometer casing.

In the last decades, numerous studies [24,25] have illustrated the possibility of using optical fiber systems for monitoring deep ground deformations. These systems have not yet found significant diffusion in real cases mainly due to the cost, measurement accuracy, and their general reliability.

In this operational and instrumental panorama, the measurement with a portable probe and operator (Figure 2a) is still widely spread, also because it allows one to reach high quality results even in a discontinuous way (periodic and non-intensive strategy). The main advantages of the manual measurement are that it allows one to measure all the length of the borehole with a very good spatial resolution and the double reading approach. With this procedure (the measurement is repeated at the same depth and resolution but with the probe rotated 180° with respect to its own longitudinal axis), it is possible to detect measurement errors and minimize sensor drifts [10,13,16,17]. The main problems are related to incorrect positioning at the established depth, low waiting for the temperature rebalance (even very different between open air and the toe of the borehole) and to gross errors in general. A further unfavorable element is the accessibility of the site, not always possible in the case of remote locations or with seasonal accessibility. In urban geo-engineering the use of inclinometers is well known and common, especially while monitoring underground structures like tunnels, retaining walls, and diaphragms [17]. Considering the expected small deformations, in comparison with landslides, it is necessary to use a system, which can assure very accurate measurements. Moreover, if the measurement is aimed to monitor the construction work advancement the system should provide high measurement frequency and high vertical discretization. As a drawback they are affected by installation constrains, especially for the automated ones, as their, even small, infrastructures are not always compatible with an urban environment such as a active road and can be subject to vandalism.

To obtain the advantages of traditional manual inclinometric measurements in an unmanned way and with remote management, a new measurement system has been developed to perform robotic inclinometric measurements in an automatic way. This paper describes the main features of the developed instrumentation by illustrating his features and by showing a series of results obtained in a real monitoring case in the field of geo-engineering. Data were acquired in 2019, during the excavations of the T3 section of the Rome Metro Line C. In particular, hundreds of measurements were analyzed in the pre-excavation period in undisturbed conditions that allowed to evaluate accuracy and repeatability by excluding the influence of significant external factors (tube deformations, interaction with excavations works, etc.).

## 2. Instrument Features and Operation Principle

**Figure 3:**
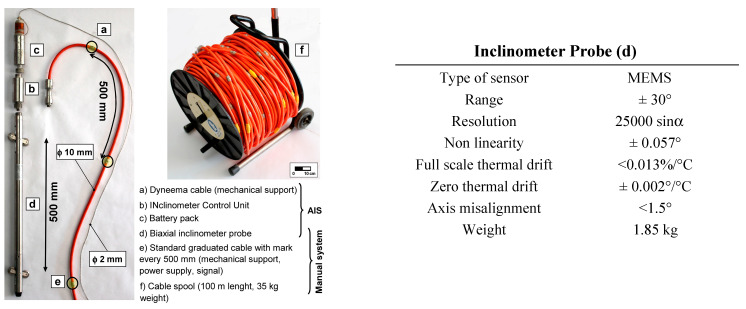
Comparison between the portable system (produced by the OTR Company, Piacenza, Italy) and AIS. In the more recent portable equipment, due to use of new digital probe, the diameter of graduated cable (e, f) was reduced to about 5 mm with 15 kg weight (for 100 m length). The probe used on AIS is the same of the portable system.

The robotized inclinometer system was designed by Research Institute for Hydrogeological Prevention and Protection (CNR IRPI) with the goal to automatize the measurement conventionally carried out by an operator (Figure 1 and Figure 2c). The main system elements (Figure 3) were preserved: the standard biaxial inclinometer probe, cable connections between the probe and ground station, and the double reading approach [26].

Some operative prototypes were developed and used in the ‘90s [27], obtaining interesting results in the field of monitoring of landslides [28,29]. Recently the system was redesigned and patented, with the following goals: modernize the mechanics, update and upgrade the electronics, and remove the conventional cable connection between probe and ground station [30].

**Figure 4:**
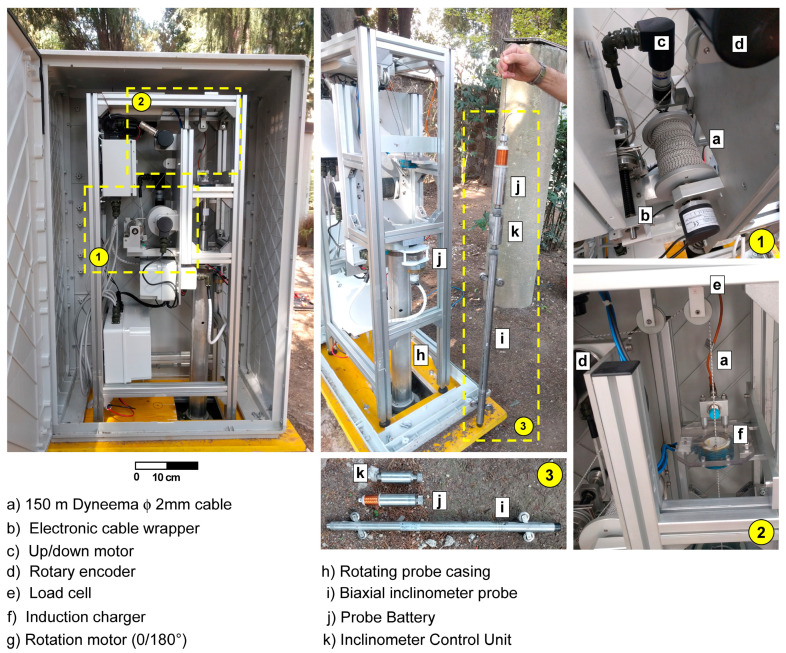
Main components of the Automated Inclinometer System (AIS) system realized by Italsensor Company, Pinerolo, Italy (www.italsensor.com).

**Figure 5:**
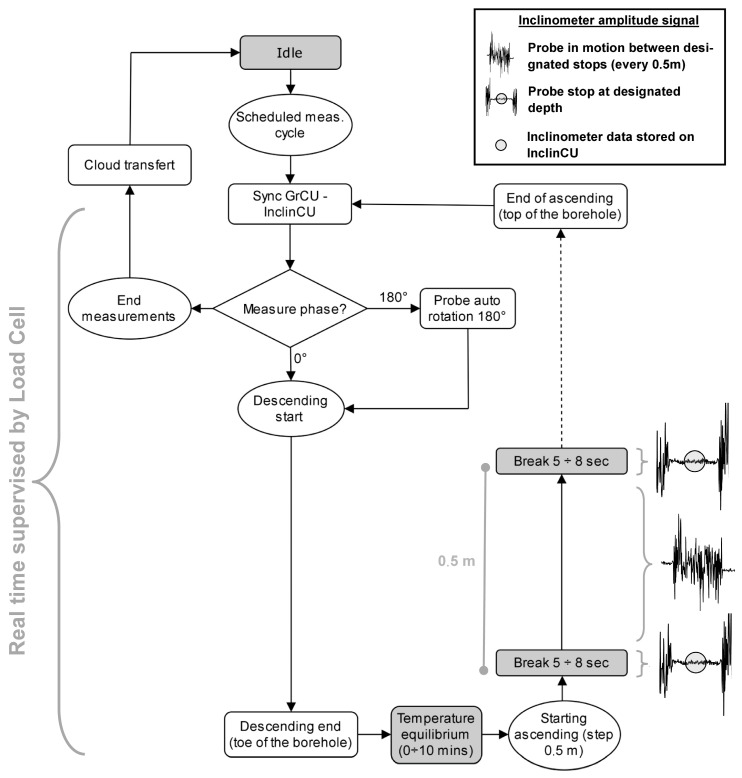
Principle of cycle measurements and signal analysis (automatic probe stop detection). From the start to end cycle, the probe is constantly weighted by the load cell (in the Ground Control Unit (GrCU)) that stops all the activities in case of lightening (obstacles in the tube, excessive deformations, etc.).

The system is composed of two main parts (Figure 4): the Ground Control Unit (GrCU) and the Inclinometer Control Unit (InclCU). The first one controls probe uplift/downlift into the inclinometer tube while the second, coupled with the probe, is the measuring part. These two components are only linked by a thin fiber cable (Dyneema^®^, φ2 mm) mechanically supporting and moving the probe in the inclinometric tube. The communications between GrCU and InclCU (setup parameters, data download) take place via radio link exclusively during the idle phase (Figure 5). During the downlift (Supplementary Material Video S2) and uplift operations, there are no communications between the systems and the InclCU is fully autonomous from power supply and signal analysis points of view. The power supply of the InclCU (and the probe) during the measurement phase is provided by the connected battery pack previously charged during the idle phase (Figure 4j).

The measurement cycle (Figure 5) is organized into two phases with the aim of performing a double reading approach. The first in the 0° position and the second with the probe rotated 180° respect to its longitudinal axis (Supplementary Material Video S1). Both phases develop with a continuous descent to the borehole bottom and then an uplift phase with steps every 500 mm with 5–8 s stops (Supplementary Material Video S3). The GrCU using a micromotor and a high precision rotary encoder (Supplementary Material Video S4) carries out these movements (Figure 4d). Since during the measurement phase no communication takes place between the GrCU and InclCU, the InclCU must be able to define autonomously when the probe is stopped at the designed depth (and ready to measure) and when instead it is moving between two consecutive designed depths (and then not allowed to measure). The InclCU continuously analyzes the signal during the uplift phase and stores the inclinometric data only when the algorithm that identifies the “stationary probe” condition is satisfied. Once the probe reaches the idle position, using a radio connection, the InclCU transmits to GrCU the measurements stored during the uplift phases (0 or 180°). In this position, the battery charge starts (Figure 4j) using a contact-less induction charging system (Figure 4f). During all the measurement phases, the measurement subsystem (probe, InclCU, battery, and cable) is constantly weighed by a load cell (Figure 4e). This system aims to identify any problems that may prevent correct uplift/downlift of the probe. The most common issue is that in the case of large deformations, the probe is no longer able to pass due to the high curvature of the tube. In these cases, the system enters in a safeguard phase and all the measurements are interrupted without “losing” the probe due to tube interlocking.

**Figure 6:**
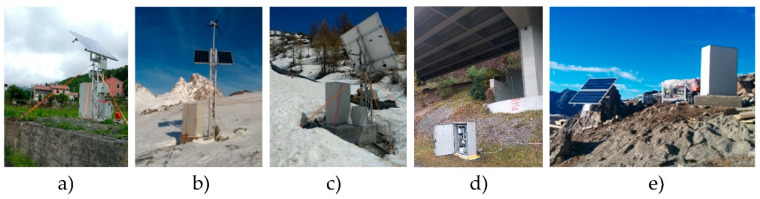
Examples of AIS installation for landslide monitoring: (**a**) Italy—Liguria region (length: 65–600 m a.s.l.); (**b**) Spain—Tena valley (length: 44–1800 m a.s.l.); (**c**) Italy—Aosta Valley region (length: 45–2300 m a.s.l.); (**d**) Italy—Trentino Alto Adige region (length: 35–400 m a.s.l.); and (**e**) Switzerland—Matter Valley (length: 50–3000 m a.s.l.).

In case of these problems, the instrument can be easily moved on another inclinometer tube without system degradation. Recently, the load cell data has also been used to automatically identify the water level inside the tube by detecting the start of the hydrostatic thrust, which causes a noticeable reduction in weight values (Figure 5). This value is not always to be considered indicative of the water table, as there should not be water circulation in the inclinometer tube. However, this parameter, taken with caution and analyzed for a long time, can be an indirect and qualitative indicator of the surrounding water level. The new prototype was successfully used in EU projects and in many experimental activities in the field of landslides monitoring in European mountain ranges [26,31,32,33] (Figure 6).

## 3. Results and Discussion

**Figure 7:**
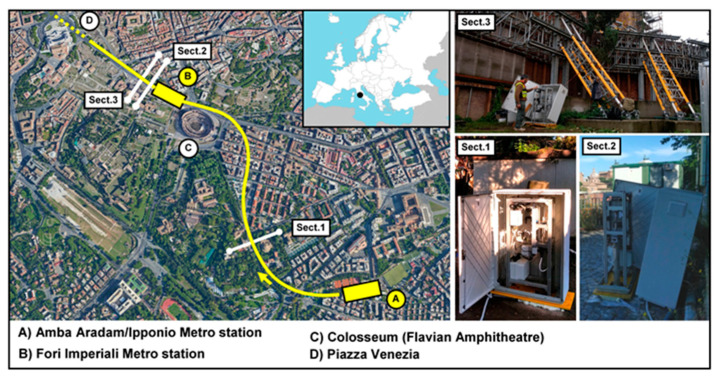
Location of test sites in Rome.

**Figure 8:**
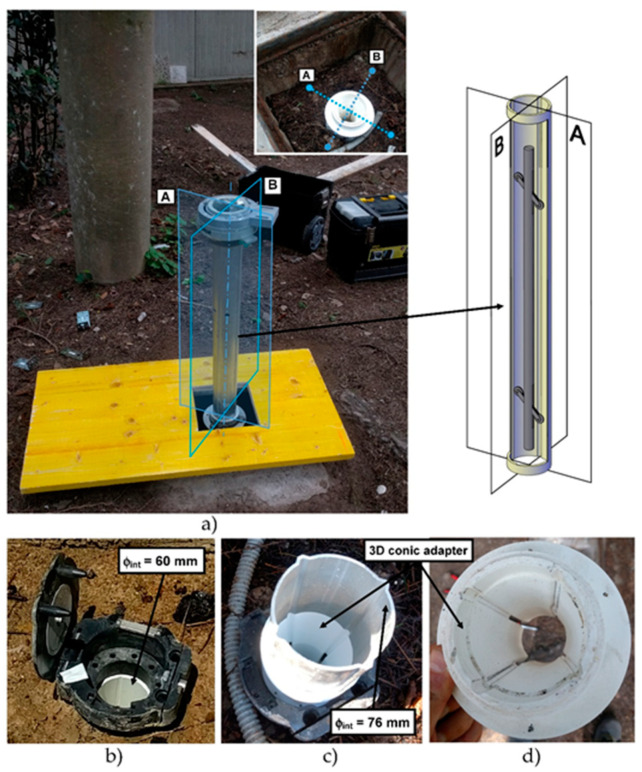
(**a**) Inclinometer probe setup during the AIS installation in each section. The A channel is represented by the plane that contain the wheels and the B channel represents the orthogonal plane; (**b**) the inclinometer casing before AIS installation; and (**c**,**d**) 3D printed conical adapter for coupling the two pipes of different diameters (ABS—62 mm and aluminum—76 mm).

In the last 5 years, the Automated Inclinometer System (AIS) has been adopted in many monitoring networks for the measurement of deep-seated ground deformations in landslide areas [31,32,33]. The data obtained from these studies confirmed the potential of the instrument in terms of precision and reliability. However, considering that the system has worked on active phenomena, it was not always simple to split the component related to instrument tolerance to the one induced by landslide activity. To overcome this limitation, numerous tests were conducted in a stable area in a 10 m tube long obtaining indications on the accuracy of the measurement [26]. After many applications in the field of landslides, we recently installed it in a historic urban on a large engineering infrastructure that began in 2019: the section T3 of the line C of the Rome metro (Figure 7 and Figure 8). The system was used on three-inclinometer tubes, realized in ABS, 62 mm diameter and lengths varying between 33 and 63 m approximately (Table 2). The analyzed measures cover a period from progressive installation in the various sections (from Section 1 to Section 3) up to about 15 days before the passing of the Tunnel Boring Machine (TBM). In anticipation of the high acquisition rate expected during the TBM passing (up 6/8 measure per day), the system was connected to the electrical network available in the construction areas. AIS was remotely connected by a 4G modem. The measurements acquired in these three periods can be considered undisturbed by the excavation or by other significant activities carried out in the soil and subsoil. Following the typical approach used in the geotechnical field, the measurements was analyzed in differential, i.e., compared to the first measurement arbitrarily chosen as a reference. The data were analyzed without the tube-spiraling correction [13,17], as it was considered a constant part and then negligible for the present study.

**Figure 9:**
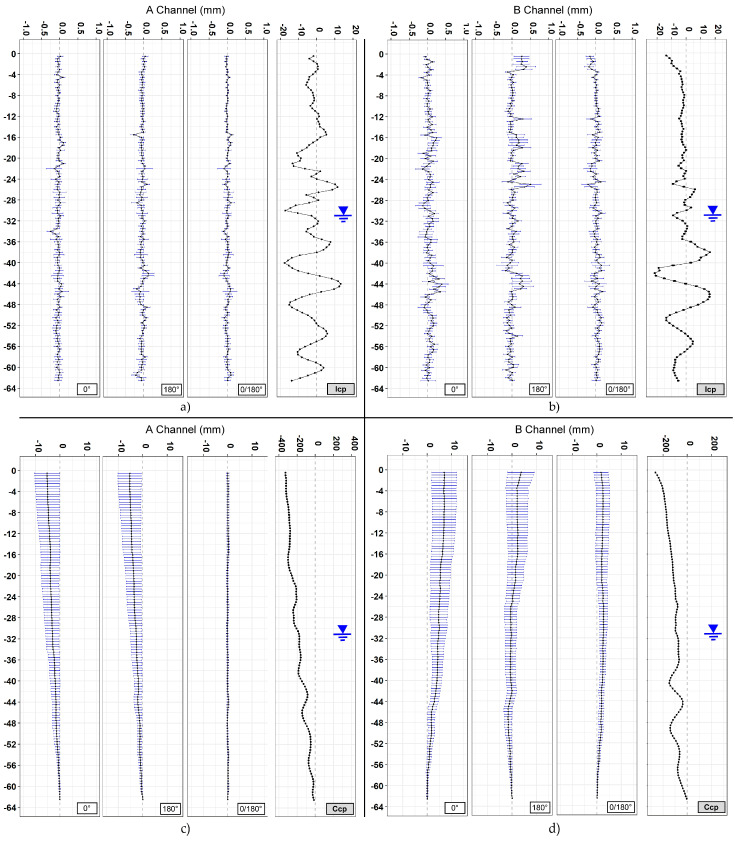
Section 2. (**a**) Incremental displacements for the A channel; (**b**) incremental displacements for the B channel; (**c**) cumulative displacements for the A channel; and (**d**) cumulative displacements for the B channel.

**Figure 10:**
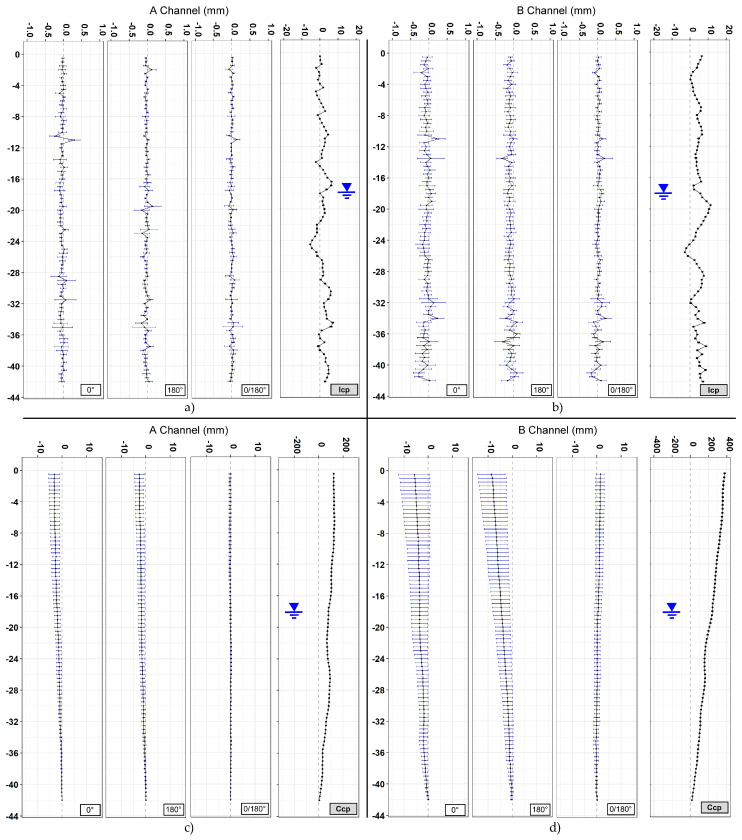
Section 2. (**a**) Incremental displacements for the A channel; (**b**) incremental displacements for the B channel; (**c**) cumulative displacements for the A channel; and (**d**) cumulative displacements for the B channel.

**Figure 11:**
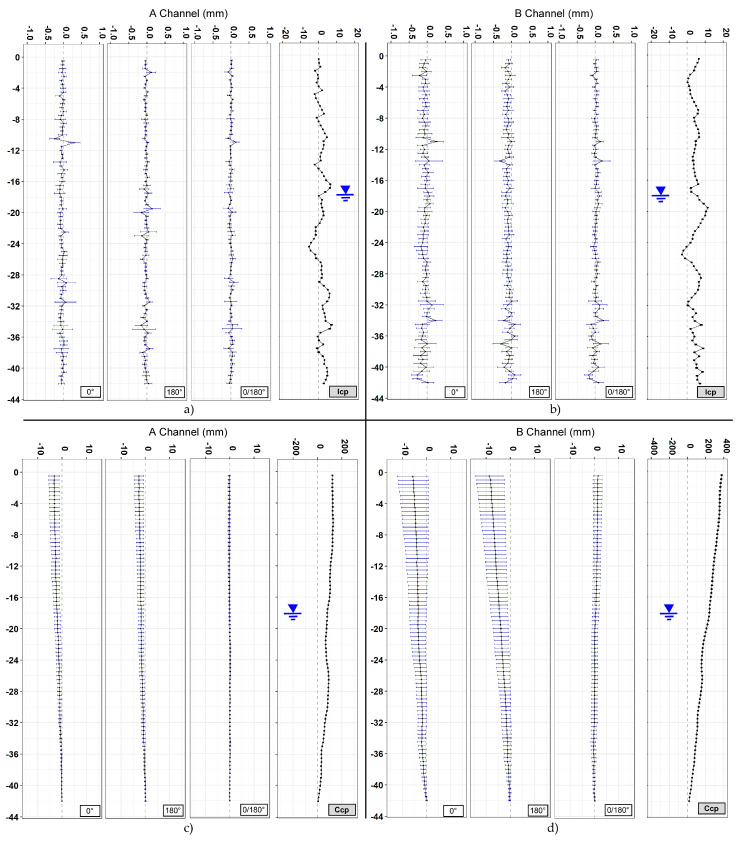
Section 3. (**a**) Incremental displacements for the A channel; (**b**) incremental displacements for the B channel; (**c**) cumulative displacements for the A channel; and (**d**) cumulative displacements for the B channel.

A total of 16 vertical profiles were plotted for each of the sections analyzed, 4 + 4 for the A channel and 4 + 4 for the B channel (Figure 9, Figure 10 and Figure 11). Since the drilling is never carried out perfectly vertically, in engineering practice all measurements are referred to the first one (first reading). To assess the overall verticality (casing profile), the measures are analyzed absolutely (non-differential) with incremental approach (every 50 cm) or in cumulative way (cumulative casing profile). The representations summarized in Table 3 include both the differential analysis from the measure chosen as the first one (difference from first reading) and non-differential analyses (first reading). Generally, the differential plots show the evolution and the changes of the observed phenomena while the first reading (non-differential) is the starting condition of the monitoring activities.

In Section one, located near the Basilica of Santo Stefano Rotondo al Celio, the largest number of measurements was taken (184 measurement cycles in about 75 days). The obtained results (Figure 9, Table 4) show an average difference for incremental displacements (in double reading) equal to 0 ± 0.07 mm for the A channel and −0.13 ± 0.12 for the B channel. As for cumulative displacement, there is an accuracy of measurement of channel A about 3 times better than channel B (−0.7 ± 0.7 versus −2.2 ± 2.6). As regards cumulative displacement, the accuracy of measurement on the A channel approximately was 4.5 times better than the B channel (0.43 versus 1.93). In Section 2, located in Via dei Fori Imperiali, 55 measurements were carried out in about 2 months. The results (Figure 10, Table 5) show an average difference for incremental displacements (in double reading) of 0 ± 0.08 mm for the A channel and 0.02 ± 0.11 mm for the B channel. Regarding the cumulative displacements, the calculated accuracy of the A channel was about 3.8 times better than the B channel (0.38 versus 1.43).

**Figure 12:**
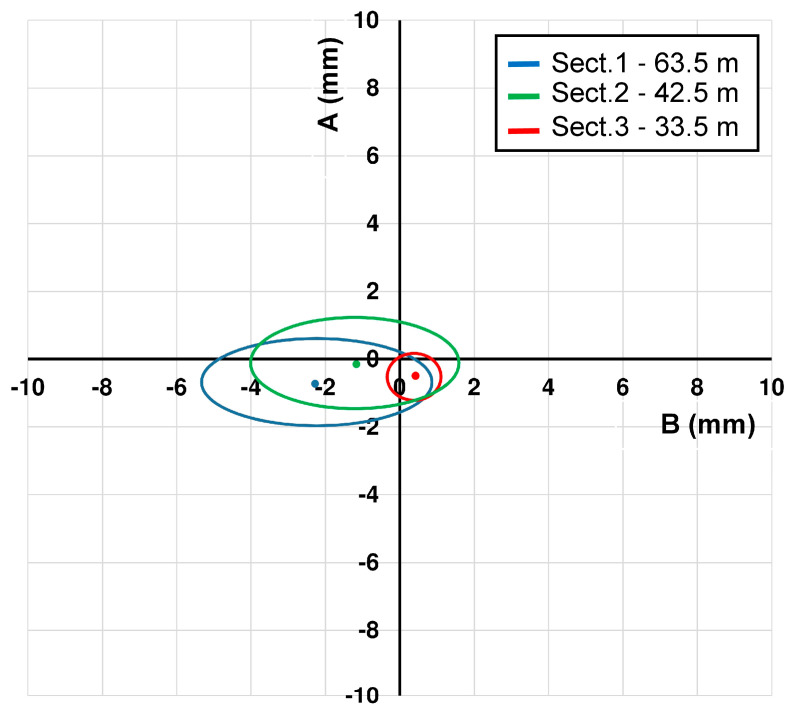
Ellipses of cumulative displacements on the top of the borehole for the three-inclinometer tube (Section 1, Section 2 and Section 3).

**Figure 13:**
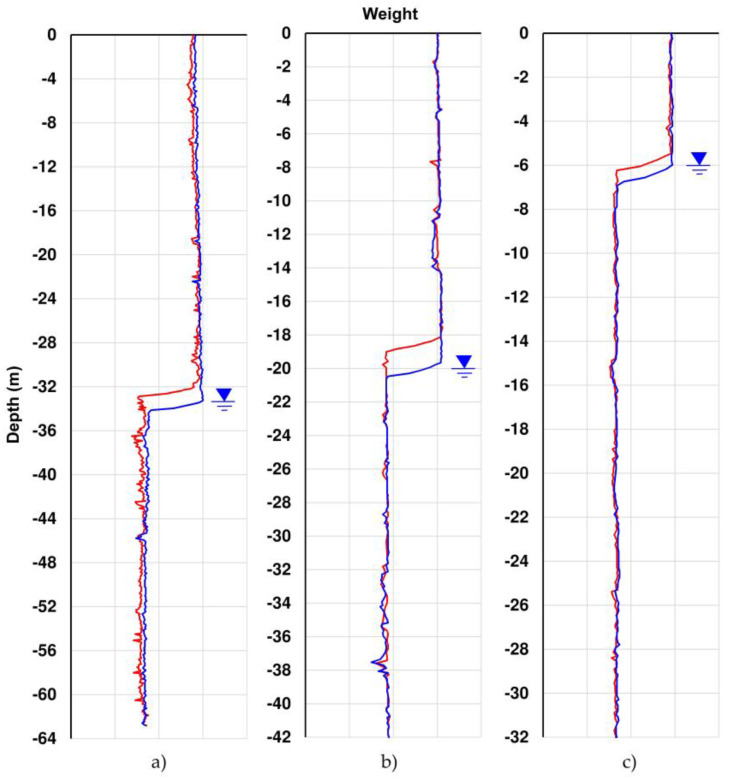
Water level measured into inclinometer tube using the load cell data (in red starting day, in blue ending day of analyzed period). (**a**) Section 1, (**b**) Section 2, and (**c**) Section 3.

The third and last section is located in Via dei Fori Imperiali near to the Basilica di Massenzio. Measurements were acquired from 5 October to 5 November (91 measurements). The obtained results (Figure 11, Table 6) show an average difference for incremental displacements (in double reading) equal to 0 ± 0.03 mm for the A channel and 0 ± 0.05 mm for the B channel. Concerning cumulative displacement (Figure 12), the calculated value of A channel accuracies was about 4 times better than the B channel e.g., 0.10 versus 0.41. Regarding the water level there were no remarkable variations during the measurements phases (Figure 13).

**Figure 14:**
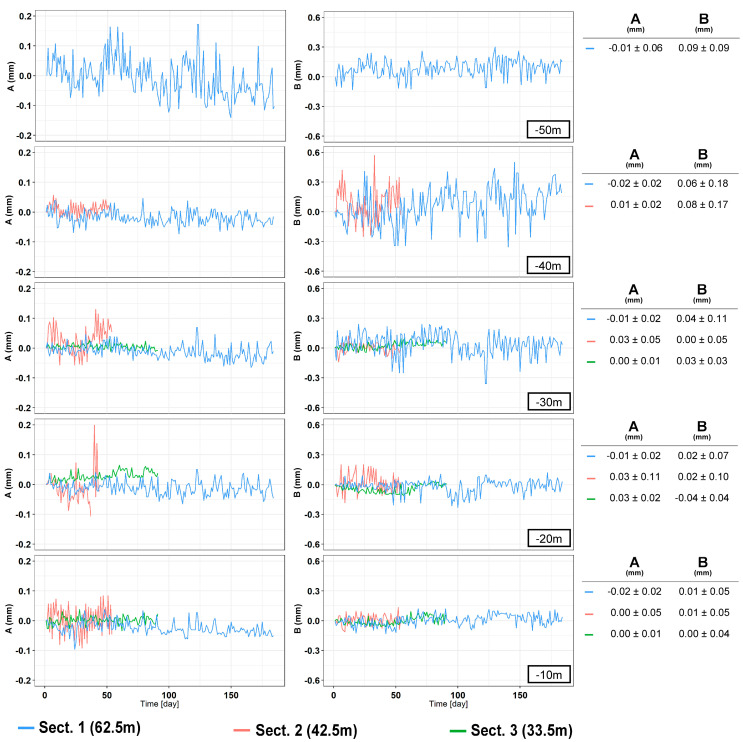
Time series of incremental displacements at different depths for each inclinometer tubes. On the right tables the statistics (mean and RMS) for each channel.

**Figure 15:**
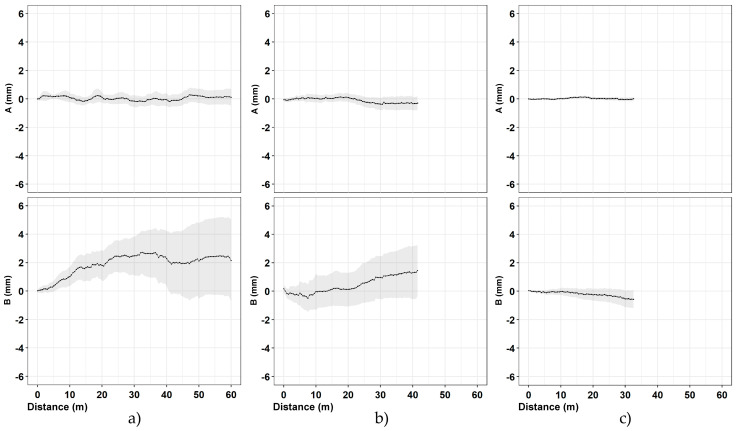
Cumulative displacements for each channel (upper frame channel A, lower frame channel B) in relation to distance from the toe of the borehole: (**a**) Section 1—62.5 tube length; (**b**) Section 2—42.5 tube length; and (**c**) Section 3—33.5 tube length. The grey area shows the error interval (RMS).

The data acquired on the three boreholes show similar indications in terms of quality between the two channels (A and B). In the field of incremental displacements, channel A is approximately 1.6 times more accurate than channel B (1.38/1.71). As for cumulative displacements, they were affected by the errors propagation (with algebraic sign) and the A channel was approximately 4 times more accurate (3.76/4.49) than the B channel (Figure 14 and Figure 15). These quantities, already observed qualitatively in previous studies [13,34,35], confirm also what has been observed in other test sites [31,32,33]. For Section 1 (Figure 9), quite wide incremental curvatures were observed for both channels. The very low diameter tube created a very strong probe/tube coupling on plane A that also induced considerable stress on the probe centering springs. On the other hand, on channel B, the slight width difference between the casing wheels’ guide and the wheel thickness could create a slight variability of inclination on plane B [13,36]. The high stress imposed by the small tube diameter on the springs combined with the high number of measurements, led to two episodes of springs rupture on the same probe (lower wheels first and, after, higher ones). These cases have never been recorded in any other monitored site even in cases of a greater number of measurements (more than 1500 cycle of measurements with the same probe and same springs). The only meaningful difference between all the other monitored sites and the three pipes in Rome is the different diameter of the inclinometer casing (ABS—62 mm in Rome, aluminum/ABS—76 mm, and all the others). This result is potentially critical in some situations and suggested a careful evaluation in the inclinometer tubes choice (of diameter in particular) and the material of the probe springs.

Analyzing the measurement repeatability at various depths (Figure 3, Figure 9, Figure 10, Figure 11 and Figure 14), measurement variability related to the depth was not clearly observed. Some differences were observed in areas probably not correctly filled or in the presence of backfilling materials such as for example 0 ÷ −10 m in Figure 10 and 0 ÷ −5 m in Figure 11. A noteworthy element for all the pipes can be observed by analyzing the absolute incremental value of inclination (a) and (b) of Figure 9, Figure 10 and Figure 11. For Section 1 (Figure 9), quite wide incremental curvatures were observed for both channels. In very limited sectors (−24 m, −42 m), slight incremental variations were observed, which corresponded to a more marked variance of the differential measurement (Figure 14, −40 m). Regarding the third tube (Figure 11), there were not appreciable variations in incremental curvature and the variance values also remained low and fairly constant. The significant incremental curvature can find a probable explanation into the compression effect that occurs during the vertical pipe installation that create small laterally bending during the pushing from the top to bottom. These types of tube shapes, which are absolutely normal in engineering practice, represent a singular point that can influence the repeatability of probe positioning and consequently the inclinometer measurement. Considering that the modern probes are extremely accurate, even a small variation of less than one millimeter in vertical positioning can induce a slight variation in inclination measurement over time. This difference, which is extremely narrow and appreciable only with an automatic and high frequency measurement approach, is usually negligible for the standard monitoring activities. As for the presence of water into the tube (Figure 13), it did not seem that its occurrence substantially impacted the measurement accuracy. However, from the preliminary analysis, the presence of water influenced the amplitude of the inclinometer signal, inducing a damping effect when compared to dry sectors.

(1) $${\mathrm{Displ}}_{A}=-0.01x\;\pm 0.01x,$$

(2) $${\mathrm{Displ}}_{B}=-0.02x\;\pm 0.04x,$$

where:

*x* = tube length (in meters);
${\mathrm{Displ}}_{A,B}$ = cumulative displacements for each channel (in millimeters);

With these boundary conditions:

Length of tube: (up to 60 m);
Inclinometer casing: ABS, 62 mm diameter;
Probe: MEMS sensor ± 30° range, standard accuracy;
Double reading approach;
No error correction.

**Figure 16:**
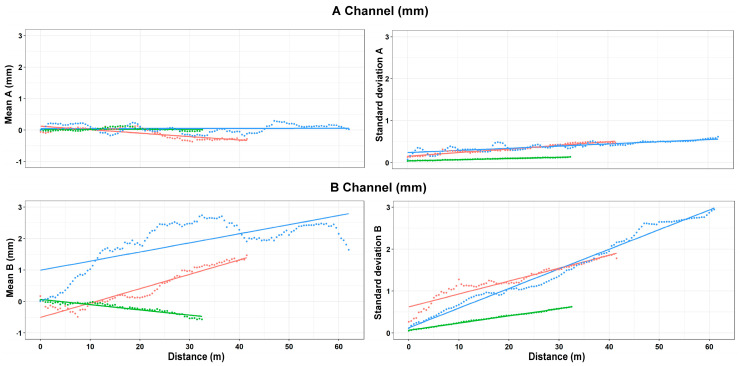
Cumulative displacements (and coupled standard deviation) as a function of the length of the borehole for the three sections observed.

The independence of the measurement variability to tube depth (Figure 15) and the algebraic sum of the incremental displacements induced an almost linear variability of the cumulative measurement accuracy (Figure 16). A synthesis attempt is illustrated in Figure 16 and in Equations (1) and (2), where a linear trend of the cumulative displacement is expressed by considering an average displacement value and a variance as a function of the tube length. This formulation, valid for the conditions illustrated in this paper, can also represent a reference formulation for measurements made by operator using a portable probe. In particular, the result calculated by 1 and 2 can be considered a reference value for maximum reachable accuracy and to define a minimum displacement, which can be considered reliable and metrologically robust. The comparison explained in the Table 7 shows the high improvement in the repeatability value when compared to very dated studies [9,10,13] but also a remarkable increase if compared to more recent research [35]. Relating to the results presented in this paper, the single errors were not analyzed individually but the results considered all the errors that characterize this type of measurement [5,10,13,36,37]. The robotic approach allows one to minimize the typical issues of the operator and to apply correctly all the best practice explained in many operation manuals such as double reading, temperature equilibrium, correct depth positioning, same probe, and same external conditions [5,10,16,17]. Referencing to Note 1 of ISO/DIS 18674-3: 2017, each of the three sections is compliant with the conditions of: (a) an identical observer, (b) an identical measurement procedure, (c) identical instruments, and (d) identical influencing quantities. Regarding the vertical repositioning at the designed depths, the elements that influence the repeatability are related to the precision of the rotary encoder and inextensibility of the cable. Concerning the encoder (Figure 4d), due to the absolute measurement with respect to the starting position and the high angular resolution (8192 point/revolution), the absolute positioning uncertainty at the *i*-th depth is less than one millimeter. As regards the cable extensibility, comparative analyses have shown negligible elongation values also in consideration of the small weight of the measuring system (probe, InclCU, and battery pack) and the weight of the cable, which increased progressively but with negligible values during the measurement phase (Figure 3, Figure 4 and Figure 5). The overall measurements carried out and presented in this paper reached approximately 45 km of inclinometer measurements considering the double reading approach (22.5 in single reading). From a qualitative point of view, the number of measurement cycles rejected was very low (3%) and the main reasons for the rejection are due to: (i) the insufficient battery level to power InclCU during the uplift and (ii) checksum values (0/180°) above the preset threshold. Regarding the environmental conditions, the system worked with temperatures varying between 8 °C in the first period (Section 1, in February) and 48 °C reached in Section 2 in August. The maximum temperature recorded in this test is the maximum that the system has ever worked on. Instead, the absolute minimum, around −25 °C, was reached during the monitoring of the Gugla landslide in the Matter Valley (Figure 6e). In addition to the breakage of the probe springs illustrated above, no significant equipment problems were recorded.

## 4. Conclusions

The paper described the robotic inclinometer system developed by the CNR IRPI for the measurement of horizontal deformations in the subsoil. In order to show the potential and the performing qualities of the system in several applications, a series of tests were conducted in the context of monitoring the new section T3 of the C line of the Rome metro. The time interval analyzed covered three sub-periods before the passage of the TBM in three sections in the monumental urban area. These periods can be considered undisturbed (from excavation activities, deformations of the subsoil, etc.) as they end at least 15 days before the excavation activities. The results obtained confirm the potential of the instrumentation and confirm what was already observed in previous studies in the field of landslides monitoring. The data acquired in real conditions and in almost undisturbed sites was fundamental to define the real performances in terms of accuracy and repeatability. The reference literature for the inclinometric measurement mainly reports real case studies in which it is not always easy to separate the instrumental precision from the perturbation component of the observed phenomena (landslide movement, excavation front, retaining walls, etc.). Furthermore, these accuracy values taken as technical standard, refer to rather dated studies (1975–2005) and with datasets related to manual measurement and the limitations connected to it. The results discussed in the paper improve the standards for the inclinometer measurement performed with a portable probe or with IPI. The reasons for these performances are mainly related to the robotic system approach, the performance of the acquisition algorithm, and the execution of the double reading.

Inclinometer measurements, born in the late 1950s, continue to be the most used approach for measuring deep-seated horizontal ground deformations. Currently, as in the last twenty years, an operator carries them out manually with a portable probe or automatically using of an inclinometer sensors string (IPI). The AIS is a modern possibility offered by a robotic high precision system that uses only one inclinometer probe for all the lengths of the boreholes. The robotic system allows one to optimize everything suggested in best practices for inclinometer measurements. The obtained results show how the accuracy of the entire measuring system (robotized probe uplift/downlift, inclinometer control unit, and probe) reached the accuracy values that are generally ascribed only to the inclinometer probe during laboratory tests. As a corollary to the presentation of the equipment and the obtained results, a general indication was formulated for the evaluation of the accuracy of the inclinometric measurement as a function of the tube length. This formulation, valid in particular conditions, can represent a reference value also for a more correct evaluation for manual and IPI inclinometric measurements, especially in the case of very low deformations.

## 5. Patent

The AIS is registered under Italian Patent UIBM 0001391881—2012.

## Figures and Tables

**Table 1:** Most used inclinometer measurement systems.

	Biaxial Portable Inclinometer Probe and Manual Operation	In-Place Inclinometers (IPI) with a String of Biaxial Sensor
**Vertical discretization**	500 mm (EU) or 2 feet	1000/3000 mm
**Temporal resolution**	Periodic, according with goals and site accessibility	1/24 meas/day
**Double reading (0–180°)**	Yes, always. It is also possible fourfold reading (0–180°) and (90–270°)	Not possible due to static position
**Probe positioning in the borehole**	The probe is positioned at the designed depth using a graduated electrical cable	Probe permanent positioned at the measurement depth
**Connection between probe and data logger**	Graduated electrical cable for sensor reading and probe support	Electrical cable for data reading (by bus system). Hard probes connection (metal or plastic) or by steel cable.
**Accuracy**	Generally high, but related to the quality of manual operations and double reading	Generally high. Some uncertainty on long term sensor stability
**Complete measure of the borehole**	Yes	Yes, if the borehole is equipped for all the length. In most cases, only some parts of the borehole are equipped by the sensors string.
**Capacity of description of thin shear bands**	The standard space is almost always sufficient to correctly describing also the thin shear bands	In case of a low number of sensors (and/or low space resolution) the shear band can be an approximate and the displacements underestimated
**Capacity of description of very large displacements**	The capacity is related to the passing of the probe into the deformed inclinometer casing	With the permanent position, is possible to measure large displacements. Many times it is not possible to extract the string sensors
**Repeatability (ISO/DIN 18674-3 2017)**	±2 mm/30 m for the main channel. Lower for secondary (transverse) channel	±2 mm/30 m for the main channel. Lower for secondary (transverse) channel
**Costs**	Low/medium and related of labor costs and periodicity of the survey	Medium/high. The costs are a function of number of sensors installed

**Table 2:** Main characteristic of the inclinometer tubes installed. The cumulative eccentricity was calculated on the top of the borehole and the incremental value is a mean for each depth (every 50 cm).

Section	Tube Length [m]	Eccentricity (Cumulative) |A|,|B| [mm]	Eccentricity (Incremental) |A|,|B| [mm]	Start ÷ End of Measures (2019)	Water Table Level [m]	Measure Duration	Number of Cycle Measures	Leading Lithology
1	62.5	328; 262	2.6; 2.1	6/03 ÷ 20/05	−31	1 h 25 min	184	Clay/sand
2	42.5	377; 397	1.5; 4.4	25/06 ÷ 30/08	−18	1 h 5 min	55	Sand
3	33.5	835; 212	1.6; 4.4	5/10 ÷ 5/11	−6	54 min	91	Sand

**Table 3:** Type of graphs plotted for each borehole.

Type of Graph	Channel	Difference from First Reading (Change)	First Reading
Incremental displacement 0°, 180°, 0/180°	A, B	√	
Cumulative displacement 0°, 180°, 0/180°	A, B	√	
Incremental casing profile (Icp)	A, B		√
Cumulative casing profile (Ccp)	A, B		√

**Table 4:** Summary of the results obtained in Section 1 (184 measures). In the first part are the incremental displacements and in the second part are the cumulative displacements.

		**SR 0°**		**SR 180°**		**DR 0/180°**		**RMS**	
**Increm.**		**mean**	**RMS**	**mean**	**RMS**	**mean**	**RMS**	**SR 0°/DR**	**SR 180°/DR**
	A	−0.04	0.09	−0.04	0.09	0.00	0.07	1.3	2.1
	B	0.06	0.15	−0.03	0.19	−0.13	0.12	1.2	1.6
		**SR 0°**		**SR 180°**		**DR 0/180°**		**RMS**	
**Cumul.**		**mean**	**RMS**	**mean**	**RMS**	**mean**	**RMS**	**SR 0°/DR**	**SR 180°/DR**
	A	−2.82	3.41	−2.93	3.18	0.05	0.43	7.9	7.4
	B	4.10	4.01	0.32	3.20	1.89	1.93	2.1	1.7

**Table 5:** Summary of the results obtained in Section 2 (55 measures). In the first part are the incremental displacements and in the second part are the cumulative displacements.

		**SR 0°**		**SR 180°**		**DR 0/180°**		**RMS**	
**Increm.**		**mean**	**RMS**	**mean**	**RMS**	**mean**	**RMS**	**SR 0°/DR**	**SR 180°/DR**
	A	−0.04	0.14	−0.03	0.09	0.00	0.08	1.9	2.2
	B	−0.07	0.17	−0.09	0.13	0.02	0.11	1.6	1.2
		**SR 0°**		**SR 180°**		**DR 0/180°**		**RMS**	
**Cumul.**		**mean**	**RMS**	**mean**	**RMS**	**mean**	**RMS**	**SR 0°/DR**	**SR 180°/DR**
	A	−1.71	1.66	−1.51	1.40	−0.10	0.38	4.4	3.7
	B	−3.07	3.80	−3.95	4.22	0.44	1.43	2.7	2.9

**Table 6:** Summary of the results obtained in Section 2 (91 measures). In the first part are the incremental displacements and in the second part is the cumulative displacement.

		**SR 0°**		**SR 180°**		**DR 0/180°**		**RMS**	
**Increm.**		**mean**	**RMS**	**mean**	**RMS**	**mean**	**RMS**	**SR 0°/DR**	**SR 180°/DR**
	A	0.01	0.04	0.01	0.03	0.00	0.03	1.6	1.3
	B	−0.02	0.07	0.00	0.08	0.00	0.05	1.5	1.6
		**SR 0°**		**SR 180°**		**DR 0/180°**		**RMS**	
**Cumul.**		**mean**	**RMS**	**mean**	**RMS**	**mean**	**RMS**	**SR 0°/DR**	**SR 180°/DR**
	A	0.18	0.21	0.15	0.21	0.02	0.10	2.1	2.1
	B	−0.56	1.5	−0.16	1.23	0.20	0.41	3.7	3.0

**Table 7:** Repeatability comparison in millimeters over a 30 m tube length between the robotic system and literature for manual measurements and IPI.

	AIS		Slope Indicator/Mikkelsen	ISO/DIN (18674-3:2017)	
	Ch.A	Ch.B	Ch.A + Ch.B	Ch.A	Ch.B
Sect.1	±0.35	±1.25	±7.8	±2	>±2
Sect.2	±0.42	±1.76			
Sect.3	±0.36	±0.54			
**Mean**	**±0.4**	**±1.2**			

## Supplementary Materials

The following are available online at https://www.mdpi.com/1424-8220/20/13/3769/s1, Video S1: Probe 180° rotation, Video S2: Probe descending phase, Video S3: Measuring phase, Video S4: Rotational encoder.
